# Faith-Based Advocacy for Family Planning Works: Evidence From Kenya and Zambia

**DOI:** 10.9745/GHSP-D-20-00641

**Published:** 2021-06-30

**Authors:** Mona Bormet, Jane Kishoyian, Yoram Siame, Ngalande Ngalande, Kathy Erb, Kathryn Parker, Douglas Huber, Karen Hardee

**Affiliations:** a Christian Connections for International Health, Alexandria, VA, USA.; b Christian Health Association of Kenya, Nairobi, Kenya.; c Churches Health Association of Zambia, Lusaka, Zambia.; d Consultant, Lusaka, Zambia, USA.; e What Works Association, Arlington, VA, USA.

## Abstract

Faith-based organizations and religious leaders can be effective family planning advocates for policy change, funding, and services. To do so, they need evidence-based knowledge, training, support within their faith communities, as well as respect for their beliefs and values.

## Key Messages

- Although it is often assumed that religion has a negative influence on family planning (FP), virtually all faith traditions support the concept of healthy timing and spacing of pregnancy.
- Given the growing evidence of the importance of religious leaders and faith-based organizations (FBOs) in shaping policy and reaching communities in support of FP, faith leaders play an important role in advocating for FP and increasing its uptake.
- FBOs are interested in expanding access to FP and increasing service delivery, yet governments, donors, and nongovernmental organizations give low priority to FBOs for financial, training, and commodity support of FP, resulting in a lack of services for underserved populations.
- Faith leaders become effective FP advocates with governments and donors through advocacy training, internal discussions to develop church positions on FP, technical information on FP, and evidence-based rationales for FP.

## EXPANDING THE ROLE OF faith-based organizations IN FAMILY PLANNING ADVOCACY

Worldwide, nearly 85% of people are affiliated with a religious faith,^1^ including in sub-Saharan Africa. Virtually all faith traditions support the concept of healthy timing and spacing of pregnancy, including access to the means for spacing pregnancies.^2^^,^^3^

It is often assumed that religion has a negative influence on family planning (FP), yet virtually all faith traditions support the concept of healthy timing and spacing of pregnancy.^2^ Interfaith support for FP exists, as shown in the Interfaith Declaration to Improve Family Health and Wellbeing, which was signed by a committee of Christian, Muslim, Hindu, and Buddhist leaders, to promote using their networks to promote healthy timing and spacing of pregnancies.^9^

Faith-based health facilities provide between 20% and 50% of health care services in countries in sub-Saharan Africa^7^^,^^8^ and are especially important for rural, remote, and marginalized populations, sometimes as the sole source of health care. A study of 95 faith-based organizations (FBOs) found that these organizations provide a range of reproductive health interventions, including FP, and link physical health with spiritual well-being.^9^ Because FBOs are embedded in the communities they serve, they can reach a range of groups with FP and reproductive health messages that are shaped by local cultures and beliefs and also provide services.^10^^–^^12^

Because faith influences health care beliefs and behaviors, religious leaders can influence health-seeking behaviors^4^^,^^5^ and their support of FP can increase its uptake.^6^ An evaluation of the Nigeria Urban Reproductive Health Initiative found that contraceptive uptake was 1.7 times higher for women exposed to FP messages from religious leaders than among women who were not, a statistically significant difference.^6^ Through a project involving FBOs and religious leaders in Kenya, religious leaders who implemented the project reported reaching nearly 700,000 people with FP messages and referring more than 87,000 clients to health facilities for FP services.^12^

FBOs are interested in expanding access to FP and increasing service delivery, yet governments, donors, and nongovernmental organizations give low priority to FBOs for financial, training, and commodity support of FP, resulting in a lack of services for underserved populations. When public facilities face stock-outs, commodities are less likely to flow to FBOs.^13^

FBOs and religious leaders are influential voices with policy makers and communities, with growing evidence that they can be strong advocates for FP.

Advocacy to increase domestic support and financing for FP, an important strategy for achieving the FP2020 goal of reaching an additional 120 million women with contraception, continues to be an important strategy under FP2030. With increasingly decentralized health systems, this work needs to be done at national and subnational levels, led by local organizations. FBOs, together with religious leaders, are influential voices with policy makers and with their communities,^14^ with growing evidence that religious leaders and FBOs can be strong and effective advocates for FP. An evaluation of the Faith to Action Network in 6 African countries identified several policy successes based on religious leaders' input in those countries.^10^ For example, in Ghana, advocacy from network members contributed to the government including FP in the National Health Insurance Act 852 in 2015.

Given people's participation in faith communities, the importance of FBOs in providing health care, and growing evidence of the importance of religious leaders and FBOs in shaping policy and reaching communities in support of FP, is there a wider role for faith leaders in advocating for FP?

Faith leaders may initially lack information and understanding about the role of FP in the health of women, children, and families and the issues facing faith-based health facilities related to contraceptive services, such as stock-outs. While faith leaders are trusted and experienced public speakers, they would benefit from gaining policy advocacy skills to reach policy makers and community members. Does having these skills and tools allow FBOs and faith leaders to play a role in advocating to national and local leaders to strengthen political and financial support for FP and to improve FP-related services?

This article describes an initiative to increase policy and financial commitment for FP and increase community support for FP through advocacy by predominantly Christian religious leaders in Kenya and Zambia in partnership with their health-related FBOs. Over three-quarters of both countries' populations are affiliated with Christian denominations,^15^ and Christian FBOs provide approximately 30% of the health services in Kenya and Zambia.^16^

## FAMILY PLANNING ADVOCACY THROUGH RELIGIOUS LEADERS PROJECT OVERVIEW

With funding from the Bill & Melinda Gates Foundation, Christian Connections for International Health (CCIH) partnered with the Christian Health Association of Kenya (CHAK) and the Churches Health Association of Zambia (CHAZ) between 2014 and 2019 to improve the policy and funding environment for FP by increasing the advocacy capacity of FBOs and religious leaders.

CCIH is a global network of Christian organizations and individuals promoting global health and wholeness from a Christian perspective. CHAK and CHAZ are the health technical arms of their church partners in their respective countries. CHAK has 521 Protestant health facilities and community programs and 67 church programs (registered churches and service delivery organizations affiliated with churches) in Kenya. CHAZ has 157 Catholic and Protestant member facilities, including church health institutions (e.g., mission hospitals, clinics, and rural health centers) and training institutions (nursing and midwifery and biomedical). CHAK and CHAZ are part of the health system and have memoranda of understanding with their governments. They play unique roles in FP advocacy and service delivery through the involvement of the church denominations that own health facilities and the religious leaders who represent them.

The language for FP advocacy requires careful attention to clarity and meaning to avoid misconceptions and misunderstandings.^3^ The project started with CCIH's definition of FP^17^:

*Enabling couples to determine the number and timing of pregnancies, including the voluntary use of methods for preventing pregnancy–not including abortion–that are harmonious with their values and beliefs.*

CHAK and CHAZ used similar criteria when recruiting religious leaders to engage in FP advocacy with their governments. Religious leaders needed to be pastors, bishops, or reverends of churches that owned or operated health facilities and were CHAK or CHAZ member institutions.

To empower them to speak up and use evidence-based arguments in support of FP, we trained 14 religious leaders in Kenya and 18 religious leaders in Zambia in advocacy, provided technical information about FP, and had discussions on the biblical support for FP. Most formal religious leaders are male; however, women's organizations in churches and women leaders have substantial influence in matters of reproductive health.

To empower religious leaders to use evidence-based arguments in support of FP, we trained leaders in advocacy, provided technical information about FP, and had discussions on the biblical support for FP.

The training was adapted from the Advance Family Planning Project's advocacy portfolio, which focuses on quick wins and helps advocates: (1) understand the policy environment; (2) make effective, evidence-based arguments, and (3) document, validate, and share results to allow for strategy revisions.^18^ CHAZ's advocacy plan noted^18^:

*CHAZ will train church leaders in the use of the quick wins strategy to ensure that long-term changes are broken down into incremental changes that combine to produce meaningful and lasting change.*

The FP sensitization used World Health Organization guidelines on contraception, which were also compatible with those of their ministries of health (MOHs). The biblical discussion was key to enabling religious leaders to discuss their theological interpretations of biblical passages with each other and agree on relevant passages from the Bible for use in publicly sharing about FP from a Christian perspective (Box 1).^19^

BOX 1 **Selected Message for Christian Communities in Support of Family Planning**

“So God created man in his own image; in the image of God he created him; male and female he created them.” Genesis 1:27

To be made in God's image is to have dignity, value, agency, and the authority to dream and plan, including planning a family and children.

“But if anyone does not provide for his relatives, and especially for members of his household, he has denied the faith and is worse than an unbeliever.” 1 Timothy 5:8

Parents must plan for their children, strive for healthy timing and spacing of pregnancies, and work to have the family they can support, consistent with their beliefs and values.

CHAK and CHAZ, with input from CCIH, developed advocacy plans in 2015, which were adapted over the life of the project to take advantage of new opportunities. In both countries, the advocacy plans called for engaging and training religious leaders, reaching out both internally to the church denominations/bodies and congregations and externally to communities through a range of media, and holding meetings with public officials to advocate for support and funding.

CHAZ is the civil society organization country lead in the health sector, through which CHAZ provides leadership by coordinating civil society organizations and facilitates linkages with the government and donors in the health sector. Through CHAZ's advocacy role in the country's health sector, they often present position papers at Zambia's health policy meetings and the Inter-agency Coordination Committee on reproductive, maternal, newborn, and child health.

Kenya's advocacy plan focused on 3 counties, following the 2010 Constitution that devolved Kenya's system of governance to the county level. Overall, advocacy was focused on raising political support and funding for FP in the counties and ensuring that resource allocation, most notably human resources and commodities, also included FBO facilities.

Zambia's advocacy plan focused on the national level and aimed to increase the national budget for FP to meet FP2020 commitments and adopt and expedite the roll-out of providing injectable contraceptives by community-based distributors. Additional advocacy tasks added included developing policy on task shifting; developing a post-2020 agenda as the FP2020 commitments ended; developing a post-2020 costed implementation plan; looking at other health financing opportunities (e.g., Global Financing Facility) and ensuring that FP services benefit from such; focusing on youth and teen pregnancies; expanding the FP method mix to include CycleBeads in both public and faith-based facilities for all people to have access; and increasing domestic FP financing to meet gaps in commodities as most of the FP budget was supported by donors.

Data for this article come from a monitoring system and tracking tools developed for the project, with quantitative findings and narrative reports compiled between 2014 and 2019. The monitoring system was based on the theory of change developed for the project (Box 2).

BOX 2 **Christian Connections for International Health Family Planning Advocacy Project Theory of Change**

The project theory of change hypothesized that:

- If faith-based organizations and religious leaders respected by communities and policy makers are trained on family planning and advocacy, underpinned by biblical teaching, and
- If they undertake evidence-informed advocacy based on an analysis of the situation with family planning services in their countries (or at the relevant subnational level), and
- If they speak out about family planning in their communities and interact with policy makers and other relevant stakeholders with terminology on family planning they are comfortable with,

then policy and financial support for family planning by policy makers will increase, along with community support for family planning.

## ADVOCACY TO CREATE A CONDUCIVE POLICY AND FINANCING ENVIRONMENT TO ENSURE FP GOAL ATTAINMENT

### Building Internal Advocacy

After attending training and before participating in external advocacy with policy makers, religious leaders identified the need for in-depth discussions within their churches to get approval. Internal advocacy included discussions with church leadership about issues such as the church's definition of FP, which methods it supports, and why it is advocating for increased support for FP important for their communities.

Some internal advocacy within the church involved the CHAK and CHAZ health technical experts. In Zambia, the Baptist Church invited a CHAZ staff member to lead a session on contraceptive methods to ensure the leadership understood and was supportive of the religious leader who was meeting with the MOH.

The religious leaders also conducted internal advocacy with their church membership to ensure consistent FP messages. To inform their communities on the benefits of FP, religious leaders also incorporated FP into their sermons and other community events. For example, an Orthodox religious leader in Kenya talked about FP during a session at his church for women and men. During a Salvation Army Radio station broadcast in Chikankata, a religious leader in Zambia talked about the need for Christians to embrace FP as a responsible way of practicing stewardship of the gift of procreation.

To inform their communities on the benefits of FP religious leaders also incorporated FP into their sermons and other community events.

### Developing Church Positions on FP

CHAZ led the process of clarifying the churches' positions on FP, another important step in preparing for external advocacy. Using the CCIH FP definition as a foundation, CHAK worked with churches at the county level and CHAZ worked with churches at the national level to develop their written positions on FP, including each church's own definition and the FP methods they find acceptable (Table 1).^20^^,^^21^ These written positions served to dispel the misconceptions that churches and other faiths do not support FP nor many contraceptive methods. Religious leaders used the position statements, along with talking points provided by project partners, in their advocacy discussions with public officials and communities.^22^

**TABLE 1.** Selected Church Positions on Family Planning in Kenya^20^ and Zambia^21^

Kenya	Zambia
**Anglican Church of Kenya Meru Diocese** Defines FP as having the number of children that one is able to care for in terms of clothing, food, shelter, and other needs. Gives its members freedom to choose a FP method. Uses materials on FP produced by the Ministry of Health and the Anglican Development Services Department. Discusses FP during seminars at the church, pastoral fellowship, and group meetings, such as Mothers Union, Kenya Anglican Men's Association, Youth Forums, and Conferences.	**Beracah Arise Bible Church** Defines FP as an arrangement between people in marriage agreeing on the number of children to have, when to have them, and how to space them. This is done to enhance good health for the women and children and for the couple to be economically stable to meet the family's needs. FP methods are decided by the couple with the help of medical personnel; the church cannot decide for the couple. Uses materials from the Ministry of Health and guidance of FP from qualified health personnel. The church team has interpreted the materials and messages with the Bible as a guide.
**Orthodox Church** Defines FP as scientific and natural methods that are used to control the size of a family and spacing between the children. Supports the use of pills, injectables, implants, intrauterine devices, condoms, CycleBeads, and natural FP. Has used PowerPoint presentations to educate members on FP at workshops, seminars, and symposiums.	**United Church of Zambia** Defines FP as having a size of children that a family can be able to look after and only having children when a couple is ready for them. Does not dictate the kind of FP methods that its members use but provides information such as referring couples to health institutions for professional advice. Uses its gatherings to invite professionals to give health talks on FP and distribute literature. Hinges on FP being for married couples, hence the promotion of abstinence to those who are not married.

Abbreviation: FP, family planning.

### Using Assessment Survey Findings to Shape Advocacy

To inform the advocacy strategy, the project conducted assessment surveys of FBO health facilities in Kenya and Zambia. The assessments reflected the FP, maternal, neonatal, and child health environment (e.g., policies, contraceptive security, training, and services provided by FBOs) and served to identify gaps to address through advocacy. In Kenya, 33 CHAK facilities were included in the baseline, and in Zambia, 41 CHAZ facilities were included.

Key barriers identified from the assessment surveys in both countries included stock-outs of supplies; lack of staff training; and lack of community knowledge about FP (unpublished reports). More use was made of the health facility-level findings in Kenya because advocacy focused on the subnational level there, whereas the advocacy in Zambia focused on the national level. Still, the assessment survey findings provided context for conversations with the religious leaders and helped direct CHAZ's original advocacy asks and efforts.

### Strengthening Ties With Other FP Partners

To increase in-country advocacy capacity, the project also fostered partnerships between religious groups and other FP actors working in Kenya and Zambia. For example, CHAK and religious leaders collaborated with the Meru County Health Management Team, MOH staff, and national and international nongovernmental organizations to strategize on creating the Meru County Costed Implementation Plan. Through CHAZ's active membership in the Family Planning Technical Working Group (FPTWG), it was asked to co-chair the first FPTWG advocacy subcommittee. Dr. Kennedy Malama, Permanent Secretary, Technical Services, Zambian MOH, stated that the FPTWG was very effective in its advocacy role.^23^ CHAZ was also tasked to be the focal point civil society organization for FP2020 in Zambia.

### Increasing Demand for FP

In addition to using their church platforms to inform people about FP and refer them appropriately, the religious leader FP champions used a variety of communications tools to reach the public, including television, radio, social media, online media, and periodicals (Supplement). The initiatives to increase demand were coordinated with other partners (e.g., FPTWG in Zambia).

**Figure:**
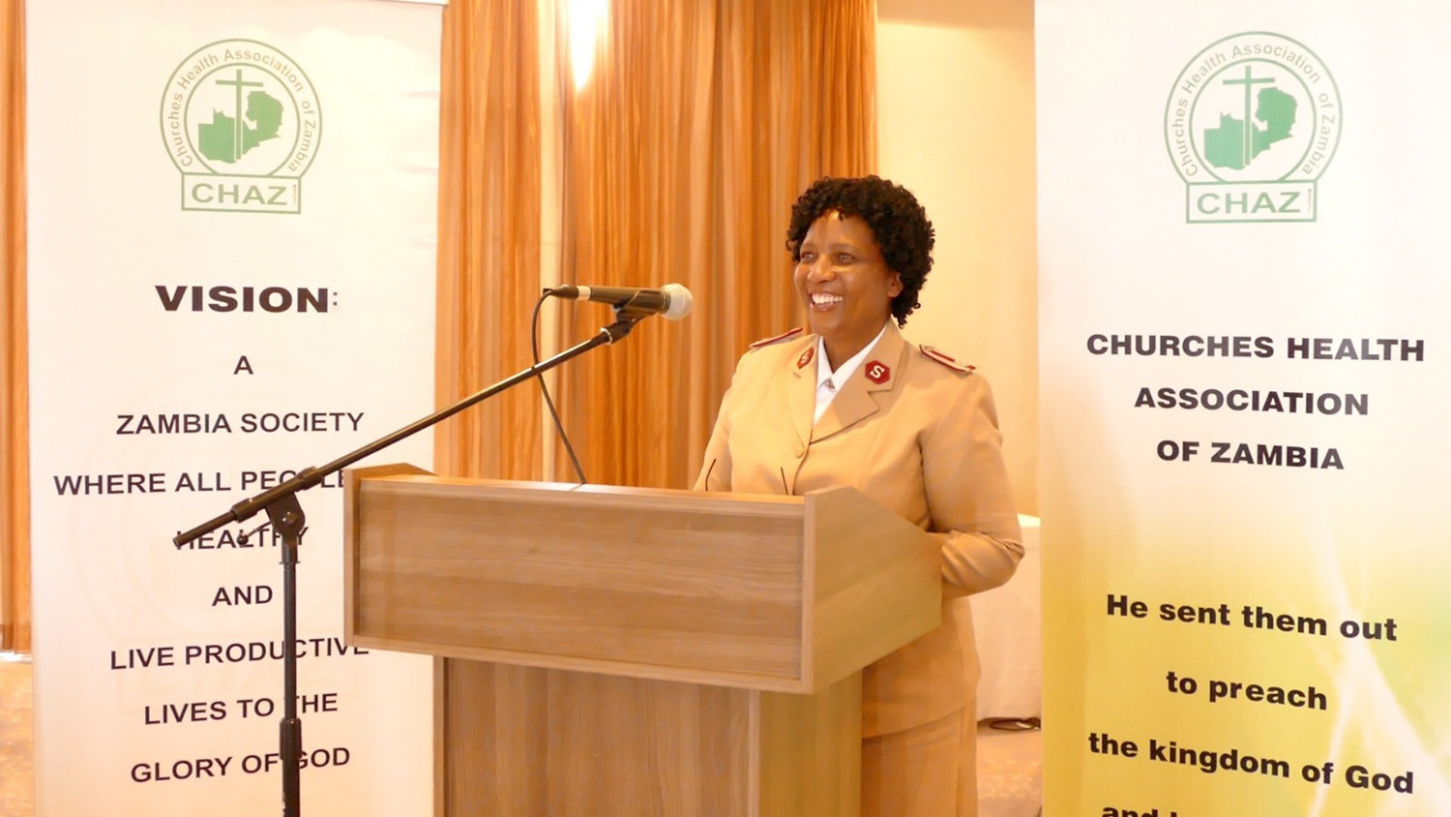
Major Angela Hachitapika of the Salvation Army Zambia Church speaks at a Ministry of Health breakfast. © 2017 Ngalande Ngalande Jr./Churches Health Association of Zambia

### Advocacy Engagement and Wins

The advocacy by the religious leaders resulted in public support of FP, political commitments for FP in both countries, commitments to developing FP costed implementation plans to increase stable financing for FP, and commitments to expand access to contraceptives through task shifting (Table 2).

**TABLE 2.** Family Planning Advocacy “Wins” Among Religious Leaders

Public Official Support for FP	A member of the county assembly in Murang'a County, Kenya, encouraged women at a rally to have children and offered payment to the pregnant women there. A religious leader FP champion met with the county assembly member and ensured that the county assembly member understood the importance of FP for healthy timing and spacing of children. Since then, the county assembly member was supportive of FP; he stopped offering money and encouraged people to have children they are able to provide for. In Zambia, at a national religious leader meeting on FP, the religious leaders urged the MOH to increase public financing for FP as opposed to being heavily dependent on external donors. The MOH recognizes the church and FBOs as strategic allies in ensuring a healthy population who should be included in a coalition to ensure they provide services that leave no one behind and achieve Universal Health Coverage.^24^
Commitment to Increasing Funding and Developing FP Costed Implementation Plans	With the county staff of Murang'a and Kiambu Counties in Kenya, CHAK conducted budget analyses, which highlighted the gaps in FP programming and what the counties could do to respond. CHAK worked with Murang'a County to develop the first-ever 5-year FP CIP in 2020, which was launched in December 2020. CHAK plans to work with the Kiambu County government to develop their FP costed implementation plan in 2021, dependent upon how COVID-19 affects staff availability toward this effort. In Meru County, Kenya, CHAK worked with others to successfully advocate to create an FP costed implementation plan, which was launched in 2018. The religious leaders continued to advocate for FP and implementation of the CIPs and for support to faith-based health facilities. The head of the Meru County, Kenya Executive Committee for Health praised the religious leaders for their work on FP and promised the county's support of FP, highlighting the importance of religious leader influence and connections. After a FP meeting attended by religious leaders and the MOH, the government of Zambia committed to increasing domestic financing, ensuring that, by 2020, its domestic contribution to FP commodities had increased to a minimum of $US1.5 million. Previously, there had been a FP line in the budget, but no funding in it. In 2017, there was a release of $US1.4 million and a release of $US2.6 million in 2018. As an indication of the need for continued advocacy and accountability, there was no release in 2019. There was also advocacy and agreement to a $US500,000 allocation for commodities.
Supply Chain Improvements for Health Facilities	Meetings between religious leaders and public officials in the 3 focus counties in Kenya resulted in verbal commitments that the MOH would help FBO facilities with contraceptive stock-outs and supply chain challenges and maintain support supervision for FP in 33 FBO health facilities in the 3 counties. FP commodities and supplies were distributed from the county stores to the FBO health facilities faster and quantities ordered were more accurate. Survey results from the 33 facilities in the 3 focus counties in Kenya in 2014 and 2017 provide an indication of the effects of the advocacy by religious leaders on FBO-run health facilities over the first 3 years of the project. Through the advocacy by religious leaders to the county governments through the CHMT, the FBO facilities have continued to receive FP commodities, with greatly reduced stock-outs in those facilities: in 2014, 40% of the facilities surveyed reported having difficulty getting FP commodities from the government, compared to 0% in 2017. Nearly all (97%) facilities reported stock-outs in 2014, compared to 20% in 2017. Advocacy with the county governments to print new FP guidelines was also successful. In 2017, the lowest level of health facilities surveyed had at least 1 copy of the FP guideline while health centers and hospitals had at least 3 copies (unpublished report).

Abbreviations: CHAK, Christian Health Association of Kenya; CHMT, county health management team; CIP, costed implementation plan; COVID, coronavirus disease; FBOs, faith-based organizations; FP, family planning; MOH, Ministry of Health.

### Reaching Youth

In 2019, the religious leaders in Kiambu County expressed concern to the County Executive Committee about the high rate of adolescent pregnancy and the high number of girls dropping out of school. The committee asked them to help educate county youth on avoiding pregnancy and HIV. As a result of this outreach and collaboration between the county and the religious leaders, county health officials spoke at church-organized events and informed the hundreds of youth who attended about teenage pregnancy and HIV, and religious leaders encouraged abstinence.^25^

CHAK and the religious leaders created a WhatsApp discussion group with the Kiambu County health staff. Conversation within this WhatsApp group continues and has been particularly useful during the COVID-19 pandemic as teenage pregnancies have increased with school closures. The county is reaching out to youth with information on preventing unintended pregnancy with the result that most girls know where to access FP services, and they are doing so. Those who became pregnant have had safe deliveries and have been supported to continue in school. The religious leaders have continued to provide messages on reproductive health and FP, and they are working with the county to form support groups for the young mothers to provide support and experience sharing as they go through motherhood and continued schooling.

**Figure:**
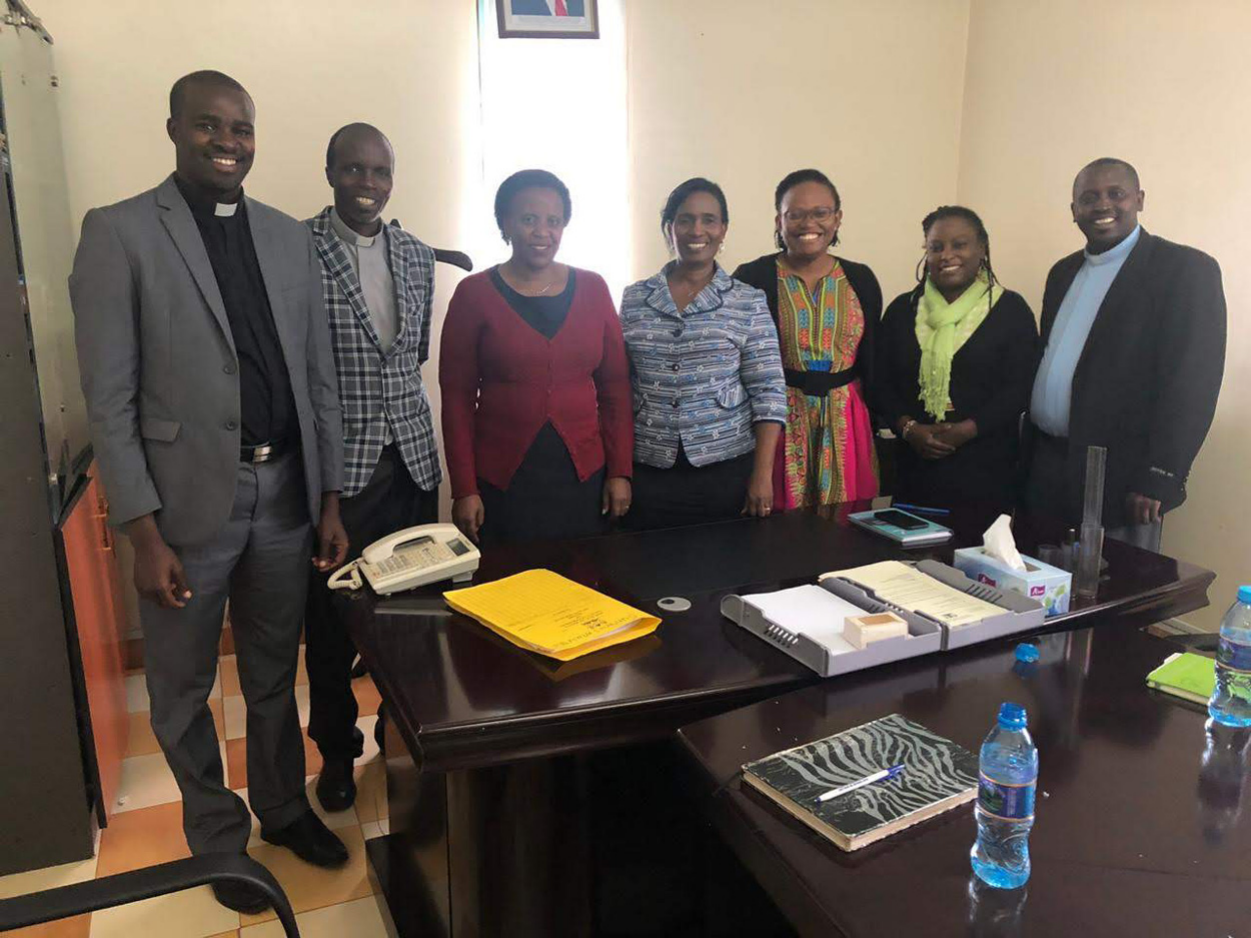
Religious leaders from Kiambu County in Kenya meet with the County Executive Committee of the Ministry of Health. © 2019 Christian Health Association of Kenya

## DISCUSSION

There have been several calls for faith community involvement in health programming.^26^^,^^27^ This article adds to the growing evidence base about the importance of FBO and religious leader engagement in FP.^6^^,^^10^^,^^12^ This project, designed to strengthen advocacy for FP by religious leaders with support from faith-based health organizations in Kenya and Zambia resulted in increased public awareness about FP and advocacy wins in both countries, even though the approaches taken in the 2 countries differed somewhat. At the same time, some aspects of implementation unfolded in unanticipated positive ways, highlighting the importance of flexibility and adaptive learning.

This project resulted in increased public awareness about FP and advocacy wins in both countries.

Several lessons learned can be drawn from this work about engaging religious leaders as advocates for FP, including: (1) religious leaders and faith-based health organizations can be strong and trusted advocates for FP; (2) internal advocacy through attention to terminology around FP and developing church positions and getting church leadership support for external advocacy is vital; (3) training in FP advocacy that includes biblical support is important for religious leaders; and (4) faith leaders need support for their modest expenses in conducting advocacy.

### Religious Leaders and FBOs Can Be Strong and Trusted FP Advocates

This article has shown that religious leaders in both countries are committed to advocating to public officials about FP and to talking about FP with communities, provided that their teachings and beliefs on FP are respected. Religious leaders are natural advocates for health issues, given their position of caring for their congregations and their connections within communities. They are respected by public officials and within communities, giving credence to their advocacy. In addition to promoting external advocacy agendas (e.g., stronger policies/more finances), religious leaders are also dedicated to promoting the health and welfare of communities and supporting FBO health facilities in service delivery.

Although attributing advocacy results to a specific initiative is challenging, the project saw shifts in the attitude and policy decisions of the MOHs in both countries that were linked with advocacy by religious leader advocates and the involvement of CHAK and CHAZ in working groups.

By grounding the advocacy in local institutions and through local religious leaders, in addition to quick advocacy wins, the project will have a long-term effect as the religious leaders continue to talk about FP to their congregations and within their communities. Engaging the religious leaders in Kenya in addressing the increase in teen pregnancy during the COVID-19 pandemic is an example of this longer-term effect.

### The Importance of Internal Advocacy Before Attempting External Advocacy

The project revealed that religious leaders need to secure approval from their broader denomination/elders before participating in public advocacy. The project facilitated developing church positions on FP and acceptable contraceptive methods. These church statements demonstrated to policy makers and the public that church bodies and denominations support FP and enabled CHAK and CHAZ, governments, and other organizations to engage more effectively with the Christian community in Kenya and Zambia.

Internal advocacy that includes sensitizing the church leaders and congregations about FP and why advocating for it is important for the church community cannot be rushed, and each church leader's processes need to be supported by technical experts and religious colleagues. It is critical to respect and work within church leaders' and FBOs' existing hierarchy and systems of protocol to achieve long-term sustainability of advocacy.

### Advocacy Training That Includes Biblical Support for FP Is Important for Religious Leaders

The religious leaders benefited from advocacy training that included technical information on FP and Biblical support for FP. The training covered the choice of methods, respected the policies of various communities, and emphasized the importance of complete and correct information, choice of methods, and voluntarism. According to an unpublished internal report, one religious leader described the training:

*If it was not for the training I had, I could not be able to talk on the radio and give information on FP and advocate for these important services, I now know that it is my responsibility to talk about FP and support it. I know I can do much more when I go back to my community.* —Religious leader, Murang'a County, Kenya

CCIH, CHAK, and CHAZ found that while there were training materials available for advocacy, advocacy on FP with religious leaders required much more relationship building and close attention to terminology. The partners published a manual documenting this integrated training approach to advocacy to help guide other faith organizations in the practical step-by-step activities that were documented from their experiences.^28^

CCIH, CHAK, and CHAZ found that advocacy on FP with religious leaders required much more relationship building and close attention to terminology.

### Religious Leaders Need Support for Advocacy

Religious leaders have many responsibilities and demands for their time, so to ensure consistency and sustainability of religious leaders' involvement, it is reasonable that they should be facilitated to participate in health advocacy activities that are supplemental to the primary role for which the church pays them. These costs would be modest given the potential continued positive advocacy impact. Costs range from transport allowances, boarding and lodging, per diem, and phone airtime, among others. Without some support and facilitation, it is unrealistic to expect religious leaders and unfunded FBOs to implement effective advocacy initiatives that would take away from their other daily responsibilities, given limited time and resources and competing priorities.

### Addressing Negative Feedback

Although the religious leaders were mostly welcomed by the public officials they sought to advocate to, in some cases they received negative feedback. In one of the counties in Kenya, for example, religious leaders received some pushback on their advocacy around county spending on health funds from the national government. Although this information should be available to the public, the county officials indicated that the religious leaders were acting as though they were “private investigators.” The religious leaders explained that, as faith leaders, they care about their communities and simply want to make sure money is allocated to support healthy families. Confidence built by the training also helped religious leaders address pushback from public officials.

## CONCLUSION

Most African Christian leaders and groups support modern methods of voluntary FP to achieve healthy timing and spacing of pregnancy and the ultimate goal of ensuring healthier mothers, children, and communities. They may have differences in terms of acceptable FP methods, and these differences must be respected. Religious leaders, with support from FBOs, can be strong and trusted advocates for FP. This project saw transformation among faith leaders in terms of their views on FP and their work to promote it. Policy changes and wider awareness in support of FP resulted from advocacy by religious leaders and FBOs. Collaborations of FBOs with national governments and partners indicate the value of faith actors' voices internally within countries to all partners and externally to global partners. These strong partnerships with governments to enhance outcomes, efficiency, and sustainability provide evidence that advocacy through FBOs makes an important contribution to common goals.

Although this project was modest in size, it demonstrated the vast potential for religious leaders to influence policy and funding for FP and other health interventions, especially ones that involve common values in support of family health and well-being. A larger number of religious leaders, equipped with evidence-based messages consistent with their religious beliefs, who are supported by their denomination leadership and faith-based technical counterparts, and given resources to cover their costs, could have an enormous and sustainable influence on local and national health policy.

## Supplementary Material

20-00641-Bormet-Supplement.pdf

## Notes

**Peer Reviewed**

**First published online:** June 9, 2021.
