# Supplementary material for: Faith-Based Advocacy for Family Planning Works: Evidence From Kenya and Zambia
Source: Glob Health Sci Pract. 2021 Jun 30;9(2):254–63. doi: 10.9745/GHSP-D-20-00641 (PMC8324188; doi:10.9745/GHSP-D-20-00641)
Supplement: 20-00641-Bormet-Supplement.pdf [file 20-00641-Bormet-Supplement.pdf]

Supplement to: Bormet M, Kishoyian J, Siame Y, et al. Faith-based Advocacy for Family Planning Works: Evidence from Kenya and Zambia. *Glob Health Sci Pract*. 2021;9(2). https://doi.org/10.9745/GHSP-D-20-00641

FAITH-BASED ORGANIZATION ADVOCACY EFFORTS TO CREATE A CONDUCIVE ENVIRONMENT TO ATTAIN FP GOALS IN KENYA AND ZAMBIA

Religious Leader Family Planning Champions Used Different Communications Channels to Inform the Public About Family Planning

<u>Radio</u>: In Kenya, a radio interview in 2016 in Kenya with 2 religious leaders from Meru and Murang'a counties and a staff member from CHAK included many call-in questions on the biblical basis of FP, and questions on common myths and misconceptions about contraceptives, and demonstrated community interest in FP to the county MOH. Religious leaders in Zambia discussed FP on 3 national radio shows in 2015 and 3 in 2016. The religious leaders addressed different components of Zambia's FP2020 commitments, e.g. investments, improving the modern method mix, increasing dialogue with religious leaders, and updating commodity security. On 2 consecutive days in 2018, a religious leader from the Beracah Christian Church who also works for ZNBC national radio in Zambia, featured the topic of FP during his morning live call-in shows. The religious leader talked about the importance of scaling up FP in Zambia. He also went out in the streets and talked about the importance of FP and engaged the public, asking them to share their thoughts on FP and religion. In 2019, an advocacy-trained religious leader from the Pentecostal Church who was an experienced media personality with programs on radio and television shared FP messages widely in the community, including to ZNBC's 9 million listenership.

<u>TV</u>: In 2016 in Kenya, a CHAK nurse went on a television show on Inoroo, a vernacular TV station, to talk about FP and display a variety of methods. About 43% of Kiambu county's residents watch this station.[1] The show's high viewership prompted the TV station to invite CHAK back to participate in another show. CHAK's Medical Director appeared on Family TV, a new Christian TV station in Kenya,[2] in which he dispelled myths about contraception and demonstrated support by Christians for FP to a wide viewing audience. CHAK was invited to appear on the network in the future with religious leaders to continue the conversation about FP. A TV interview on WERU TV (with a population reach of over 11 million people)[3] in 2019 with religious leaders from 2 churches in Meru County in Kenya[4] illustrated how FP protects the health of mothers and children and is consistent with Christian values and facilitated a direct appeal from the religious leaders to the county government to take FP seriously.[5]

<u>Opinion pieces</u>: In 2019, 2 opinion pieces by religious leaders supporting FP appeared in *The Star* in Kenya, with a circulation of over 15,000. A cleric at the Presbyterian Church of East Africa in Murang'a county made the point that "the words Bible and birth control are not contradictory. The Bible can teach us a lot about FP; a husband and wife should plan their

families because God also makes plans".[6] Also, a reverend from the Methodist Church in Meru County, noting personal experience with women in her congregation dying from causes related to pregnancy, wrote, "I urge Christians to plan and care for their families, and I urge our policy makers in Kenya to make FP a priority. The lives of women and children depend on it".[7] In 2019, an advocacy-trained religious leader from the Pentecostal Church in Zambia who was an experienced media personality with programs on radio and television shared FP messages widely in the community. Through multiple channels, the religious leader reached multiple audiences in the community along with ZNBC's 9 million listenership.

Other Platforms: Between 2018-2019, 14 CHAK-trained religious leaders in Kenya spoke about FP in sermons, at church committee meetings for men and women, seminars, in their church medical facilities to antenatal and postnatal mothers, and public barazas. Over an 8-month period in 2019, the religious leaders in the 3 counties shared 264 messages, including that FP helps protect family health; demystifying myths; scriptural support for FP; God is a planner, hence planning is good; encouragement to visit health professionals for FP information; and the role of men in FP. The religious leaders reported that they reached an estimated 14,500 people in the 3 counties.

REFERENCES

1. Ngari F. *New Vernacular TV Viewership Habits: A Case of Inooro TV.* Master's thesis. University of Nairobi; 2019. Accessed May 3, 2021. http://erepository.uonbi.ac.ke/handle/11295/108439
2. Okumu J. *Family Matters*: Family Planning. FamilyTV. January 25, 2018. Accessed May 3, 2021. https://familymediaonline.com/video_listing/family-matters-family-planning-25th-january-2018/
3. About WERU media. WERU TV. Accessed May 3, 2021. http://www.werutv.co.ke/weru-media/
4. Uciari K. Meru County Religious Leaders on WERU TV. YouTube. July 25, 2019. Accessed May 3, 2021. https://www.youtube.com/watch?v=fUsWvIhFt_Y&feature=youtu.be
5. Christian Health Association of Kenya (CHAK), Christian Connections for International Health (CCIH). *Kenya: Kenya Faith Leaders Reach a Wide Community with Family Planning Advocacy. Case Study.* CHAK, CCIH. Accessed May 3, 2021. https://www.ccih.org/wp-content/uploads/2017/09/CAFPA-Case-Study-CHAK-Year-2.pdf
6. Kariuki M. Religious leaders have active role in promoting family planning. *The Star*. November 16, 2019. Accessed May 3, 2021. https://www.the-star.co.ke/opinion/star-blogs/2019-11-16-religious-leaders-have-active-role-in-promoting-family-planning/
7. Mberia C. Family planning matter of life and death for Kenya's women. *The Star*. December 8, 2019. Accessed May 3, 2021. https://www.the-star.co.ke/siasa/2019-12-08-family-planning-matter-of-life-and-death-for-kenyas-women/
